# Construction of a Dataset for All Expressed Transcripts for Alzheimer’s Disease Research

**DOI:** 10.3390/brainsci14121180

**Published:** 2024-11-25

**Authors:** Zhenyu Huang, Bocheng Shi, Xuechen Mu, Siyu Qiao, Gangyi Xiao, Yan Wang, Ying Xu

**Affiliations:** 1 College of Computer Science and Technology, Jilin University, Changchun 130012, China; zhenyuh19@mails.jlu.edu.cn (Z.H.); xiaogy19@mails.jlu.edu.cn (G.X.); 2 Systems Biology Lab for Metabolic Reprogramming, Department of Human Genetics and Cell Biology, School of Medicine, Southern University of Science and Technology, Shenzhen 518055, China; shibc22@mails.jlu.edu.cn (B.S.); m250921296@gmail.com (X.M.); 12310427@mail.sustech.edu.cn (S.Q.); 3 School of Mathematics, Jilin University, Changchun 130012, China

**Keywords:** Alzheimer’s disease, lncRNAs, splicing variants, Fenton reaction, metabolic reprogramming

## Abstract

Accurate identification and functional annotation of splicing isoforms and non-coding RNAs (lncRNAs), alongside full-length protein-encoding transcripts, are critical for understanding gene (mis)regulation and metabolic reprogramming in Alzheimer’s disease (AD). This study aims to provide a comprehensive and accurate transcriptome resource to improve existing AD transcript databases. **Background/Objectives**: Gene mis-regulation and metabolic reprogramming play a key role in AD, yet existing transcript databases lack accurate and comprehensive identification of splicing isoforms and lncRNAs. This study aims to generate a refined transcriptome dataset, expanding the understanding of AD onset and progression. **Methods**: Publicly available RNA-seq data from pre-AD and AD tissues were utilized. Advanced bioinformatics tools were applied to assemble and annotate full-length transcripts, including splicing isoforms and lncRNAs, with an emphasis on correcting errors and enhancing annotation accuracy. **Results**: A significantly improved transcriptome dataset was generated, which includes detailed annotations of splicing isoforms and lncRNAs. This dataset expands the scope of existing AD transcript databases and provides new insights into the molecular mechanisms underlying AD. The findings demonstrate that the refined dataset captures more relevant details about AD progression compared to publicly available data. **Conclusions**: The newly developed transcriptome resource and the associated analysis tools offer a valuable contribution to AD research, providing deeper insights into the disease’s molecular mechanisms. This work supports future research into gene regulation and metabolic reprogramming in AD and serves as a foundation for exploring novel therapeutic targets.

## 1. Introduction

The vast majority of the transcriptomic data analyses of human diseases focuses on expression data of full-length transcripts of protein-encoding genes. However, such data may not be adequate for full elucidation of the disease drivers and key mechanisms as demonstrated in studies of autism spectrum disorder (ASD), bipolar disorder (BD), and schizophrenia (SCZ) [1]. Inspired by such studies, we present here a computational framework for accurate identification of all expressed transcripts of protein genes, namely all identifiable full-length and splicing isoforms, and of long non-coding RNA genes or lncRNAs, and demonstrate how the splicing variants and lncRNAs could substantially add to our understanding of disease drivers and development of Alzheimer’s disease (AD).

AD is the most common form of dementia, characterized by progressive decline of cognitive capability as a result of massive neuronal cell death, with no effective treatment currently available [2]. The most extensively studied features of AD include amyloid-beta (Aβ) plaque deposition and the formation of Tau protein neurofibrillary tangles (NFTs) [3,4]. However, recent research has revealed that AD is also associated with fundamental changes in cellular biology and chemical conditions. These include (1) increased extracellular acidification and intracellular alkalinization [5,6]; (2) dysregulated zinc and copper metabolism [7]; (3) chronic inflammation and elevated oxidative stress [8,9]; and (4) heightened neurotoxicity [10,11].

While RNA-sequencing (RNA-seq) data have been generated from postmortem brain samples of both healthy individuals and those with AD [12,13,14], published studies predominantly rely on full-length transcripts of protein genes [13,14,15]. However, similar to the aforementioned studies on other diseases, some recent works on AD and pre-AD MCI (mild cognitive impairment) have shown that inclusion of lncRNAs and splicing variants in AD studies can considerably improve the level of understanding about the disease biology [16,17,18].

Our first goal here is to derive all the splicing isoforms and lncRNAs expressed in MCI and AD tissues based on the raw RNA-seq reads available in the public domain, along with their expression levels. To accomplish this, we have collected raw RNA-seq reads from three public datasets: (i) data generated from the dorsolateral prefrontal cortex region in the ROSMAP cohort [12]; (ii) datasets from regions 22 and 36 of the temporal lobe and from regions 10 and 44 of the frontal lobe in the MSBB database [13]; and (iii) the GSE95587 dataset [14]. De novo assembly of these RNA reads were conducted using StringTie (version 2.2.1), currently the best-performing assembly software for transcripts publicly available [19]. Then, functional annotation of the assembled transcripts was conducted through mapping onto GENCODE for functional annotations of the newly assembled transcripts in support of MCI/AD studies.

Compared to the publicly available transcriptomic data for MCI and AD tissues, our de novo assembled dataset is 1.89 times larger than the currently available datasets along with having 1.845 times more functionally assigned transcripts, enabling functional studies of AD with much expanded scope and higher depth. To the best of our knowledge, this work represents the first effort to build the most comprehensive transcript-level dataset for MCI and AD studies.

## 2. Materials and Methods

### 2.1. Quality Control and Preprocessing

All raw reads were downloaded from the ROSMAP cohort (https://www.synapse.org/Synapse:syn3219045, accessed on 1 August 2020) [20]. After applying sample selection criteria, the dataset included transcriptomes from 146 mild cognitive impairment (MCI) cases, 193 Alzheimer’s disease (AD) cases, and 189 region-matched controls. To ensure high data quality, we filtered out low-quality reads from samples in the DLPC (dorsolateral prefrontal cortex) brain region of the ROSMAP cohort, resulting in approximately 2.11 TB clean bases. Specifically, for each sample, at least 90% of the reads were required to have a quality score at least Q30 (error rate ≤ 0.1%). SOAPnuke (version 2.X) was used to filter out reads with GC distribution fluctuations exceeding 10% [21]. Clean reads were mapped to the rRNA database using RSEQC (version 5.0.4) [22] to remove rRNA-derived reads. Sequencing randomness and gene saturation were also identified and filtered out using RSEQC. Library preparation varied by batch: Batch 1 libraries were prepared at the Broad Institute using a strand-specific dUTP method with poly-A selection and sequenced on the Illumina HiSeq platform with 101 bp paired-end reads, achieving 150 million reads for the first 12 samples and 50 million for the rest. Batch 2 libraries were prepared using a stranded RNA-Seq protocol with RiboErase, targeting 30 million reads per sample on the Illumina NovaSeq 6000 with 2 × 100 bp reads. Batch 3 libraries were constructed from frozen brain tissue using a modified TruSeq stranded protocol to favor larger fragments, achieving 40–50 million reads per sample on NovaSeq 6000 with 2 × 150 bp reads.

### 2.2. Genome Mapping, Transcript Assembly, and Quantification

To reduce false positives caused by repetitive sequences in the reference genome, we used RepeatMasker (version 4.1.7) [23], RMBlast (version 2.14.1) [24], and TRF (version 4.09.1) [25] to mask repetitive regions. After quality control, the remaining reads were aligned to the repeat-masked reference human genome (GENCODE version 34) using HISAT2 (version 2.7) [26] with default parameters. StringTie was used to assemble transcripts [27], and the “merge” option was applied to unify all output files into a single transcriptome. Gffcompare [28] was used to compare the assembled transcriptome (in GTF format) with reference annotations to obtain the genomic location of each transcript. Transcript expression levels were quantified in transcripts per million (TPM) using Ballgown [29]. The clinical information for the three datasets, as well as the genome FASTA file and the annotation of the assembled transcripts, is available in Supplmentary Data S1.

### 2.3. Identification and Classification of LncRNAs

To identify lncRNA genes, we employed a two-step approach involving protein-coding potential evaluation followed by lncRNA classification.

**Step 1: Protein-coding potential evaluation.** Transcripts were initially filtered based on length and exon count, with only transcripts consisting of at least 200 nucleotides and having two exons or more retained for further analyses [30]. Their protein-coding potential was assessed using five computational methods: CNCI v1.0 [31], CPAT v2.0.0 [32], CPC v0.1 [33], CPPred v1 [34], and PLEK v1.2 [35]. Transcripts were classified as lncRNAs only if all five methods all predicted them as non-coding.

**Step 2: LncRNA classification.** New transcripts that were not annotated in GENCODE [36] were categorized based on their genomic location as follows:

- “i”: transcripts entirely within an intron;
- “o”: transcripts that have some overlap with known genes but do not fully overlap; they may partially span introns and exons;
- “u”: transcripts in intergenic regions; and
- “x”: transcripts that fully overlap with the exons of a reference gene, indicating some degree of overlap with known transcript structures.

Additionally, all identified lncRNAs were further classified into intergenic, antisense, or intronic lncRNAs based on their genomic positions.

### 2.4. Differential Expression and Pathway-Enrichment Analyses

Differential expression analyses were performed to identify differentially expressed transcripts (DETs) between (MCI ∪ AD) samples and controls using the “DESeq2” function [37]. Transcripts with a fold change (|FC|) ≥ 1.3 and a false discovery rate (FDR) < 0.05 were considered differentially expressed, while transcripts with normalized read counts below 1 were excluded.

The identified DETs were subject to pathway-enrichment analysis against KEGG, REACTOME, and GO Biological Process databases. Only pathways with an enrichment adjusted *p*-value < 0.05 were considered for further study. The identified DETs and enriched pathways are available in Supplmentary Data S2.

### 2.5. Identification of Splicing Isoforms

Assembled transcripts were mapped onto the reference genome (GENCODE v34) using STAR (v. 2.7.10b), which generates BAM files. Then rMATS-Turbo was run on the BAM files using the following parameters: “—chimSegmentMin 2—outFilterMismatchNmax 3—twopassMode Basic—alignEndsType EndToEnd—alignSJDBoverhangMin 1—alignIntronMax 299999”.

We specified the read type, length, and strand specificity with “-t paired —readLength 101 —variable-read-length —libType fr-firststrand”. rMATS was used to identify splicing events with *p*-values < 0.05 and classify them into one of the following five types: SE (skipped exon), A5SS (alternative 5′ splice site), A3SS (alternative 3′ splice site), MXE (mutually exclusive exon), and RI (retained intron). Then, one more round of filtering was conducted to remove splicing events with |ΔPSI| > 0.05, where PSI is for Percent Spliced In [38], used to measure the proportion of transcripts that include a particular exon or splicing event compared to the total number of transcripts.

### 2.6. Gene Set-Enrichment Analyses for Individual Samples Using Gsva

To perform gene set-enrichment analysis (GSEA) on individual samples, we used the “gsva()” function in R with the “ssgsea” method [39]. This approach ranks genes based on their expression levels and calculates the enrichment score for each gene set, where a positive score indicates that genes in the set are highly expressed, while a negative score indicates otherwise.

### 2.7. Regression Analyses

To assess the co-expression level between an individual gene g and a gene set M, we employed a principal component-based approach. Principal components (PCs) were calculated using the expression levels of the genes in the set across the specified samples. PCs that can collectively explain at least 60% of the cumulative variance were retained [40]. Then, a linear regression of the expression level of g was conducted against all the retained PCs, namely

$$e\left(g\right)=\sum_{i}\beta_{i}PC_{i}+\beta_{0}$$

where {$\beta_{i}$} are parameters to be determined through minimizing |$\beta_{0}$| [41].

### 2.8. Prediction of Target Genes and Pathways Regulated by LncRNAs

LncRNAs can regulate gene expressions either in their genomic neighborhood (cis-regulation) or at distant locations (trans-regulation) [42,43]. Cis-regulated target genes of a lncRNA were predicted based on locational proximity within 100 kb upstream or downstream of the lncRNA if co-expressed with the lncRNA [44,45]. Trans-regulated genes were predicted based on sequence complementarity and co-expression between the lncRNA and the target genes, using RIsearch [46]. Prediction criteria also included the free energy of the bound structure between the lncRNA and each DNA sequence it binds (energy < −10) [47].

A lncRNA was predicted to be a regulator of a pathway if (1) an optimal regression model of the lncRNA against the PCs of the pathway yielded a high R² value with an adjusted *p*-value < 0.05 and (2) the lncRNA was predicted to be a cis or trans regulator of some genes in the pathway, with a Spearman correlation coefficient |r| > 0 and an adjusted *p*-value < 0.05.

### 2.9. Multi-Class Classification Analysis Using LGBM

A multi-class classification was constructed using LightGBM (LGBM, version 4.5.0) [48] to classify a tissue sample as a normal, MCI, or AD sample based on the gene expression data of a pre-defined gene set. The classification performance of the gene set was evaluated using the ROC curve and the AUC value, without feature elimination.

### 2.10. Statistical Analyses

All statistical analyses were conducted using R (version 4.3.2) and Python (version 3.11.9). The specific statistical tests applied in each experiment are described in the figure legends. Unless stated otherwise, non-parametric tests were used, and a *p*-value threshold of <0.05 was considered statistically significant.

## 3. Results

### 3.1. Creating a Transcriptomic Dataset for Alzheimer’s Disease

**Figure 1:**
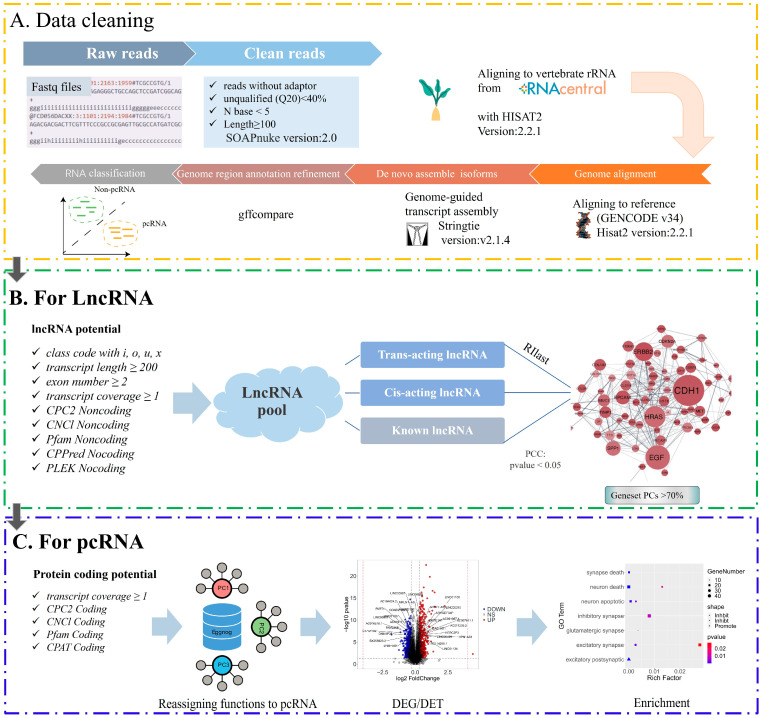
Bioinformatics analysis workflow in our study. (**A**) The orange box represents data quality-control criteria, including quality control of reads, filtering of rRNA reads, transcript assembly, and transcript type identification. (**B**) The green box represents the criteria for LncRNA identification and analysis, considering the relationship between a transfrag and the closest reference, transcript length, exon number, read coverage, five non-coding potential predictions, and target gene prediction. (**C**) The blue box represents the criteria for protein-coding RNA identification and analysis, including four protein-coding potential predictions, read coverage, functional reassignment, differential transcript analysis, and functional enrichment.

We selected 639 samples from the DLPC region in the ROSMAP database [12], which is an earliest region affected by AD [15]. Then, a filtering process was conducted on these samples to remove samples meeting the following criteria: samples diagnosed as another form of dementia by itself or in combination with AD. Then a quality control (QC) step was executed to remove samples with low quality as defined in Section 2.1, giving rise to 528 samples (Figure 1A).

The raw RNA reads of each sample were assembled to full transcripts and mapped onto the reference human genome, using the methods in Section 2.2. Then all expressed lncRNAs and splicing isoforms were identified and extracted using the methods given in Section 2.3 and Section 2.5 (also Figure 1B,C).

For lncRNAs, we performed the following: (1) identification of novel lncRNAs and their classification as described in Section 2.3; (2) regulatory target prediction for each lncRNA; and (3) pathways possibly regulated by each lncRNA, using the methods in Section 2.8.

For each splicing variant, we conducted functional (re-)annotation as follows: we searched the protein-sequence database [49] to find the closest homolog, in humans or another organisms, that has no other transcript in our assembled dataset being assigned to its function—this guarantees no two splicing variants being assigned to the same function. The following summarizes the transcripts we assembled (Table 1):

The computer code for data generation is available at https://drive.google.com/drive/folders/17CWxRNx1Cdm17IIb_d8_lyg12VrRohTx (accessed on accessed on 21 November 2024).

### 3.2. Information Derivation from the Assembled Transcripts

To explore how alternative splicing events and isoform usage differs between normal and MCI/AD samples, we first analyzed the genomic distribution of all the assembled transcripts.

**Figure 2:**
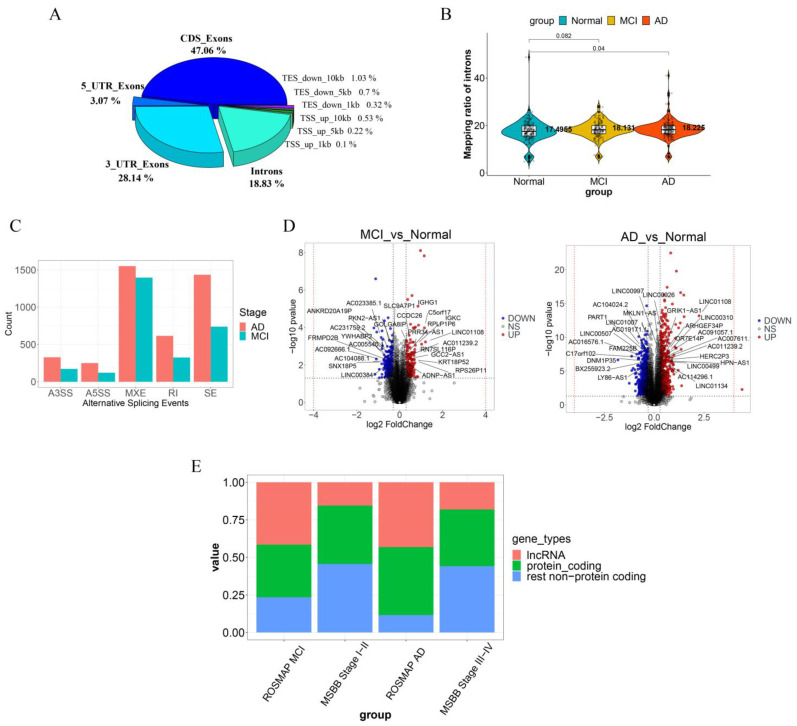
Statistical characteristics of the AD dataset. (**A**) Distribution of reads across different genomic regions. (**B**) Intronic alignment rates across normal, MCI, and AD groups. (**C**) Number of genes affected by five significantly different alternative splicing events in the MCI and AD groups compared to the normal group. (**D**) Differential gene statistics between MCI, AD, and normal groups with significantly different non-coding protein genes specifically labeled. (**E**) Composition of differential gene types across different stages in the ROSMAP and MSBB datasets.

**Genomic locations of the assembled transcripts**: We examined the distribution of sequencing reads across different genomic locations. We note that, in both normal and disease samples, 47.06%, 28.14%, 18.83%, and 5.97% of the RNA reads were mapped to the coding sequence (CDS), introns, the 3′ untranslated regions (UTRs), and the upstream regions (10K bases) of transcription start sites (TSSs) (Figure 2A).

When looking the two disease groups, MCI and AD, separately, some differences are observed. We note that (i) 18.131% and 18.225% of MCI and AD are mapped to intronic regions and (ii) 28.596% and 27.8825% of MCI and AD are mapped to 3′ UTR regions.

Further examination of read coverage across different disease groups showed notable differences. Specifically, intronic coverage progressively increases from the normal to AD samples, with significant differences: MCI samples vs. normal controls having a *p*-value 0.082, while the AD samples vs. normal controls having a *p*-value 0.04 (Figure 2B). For the 3′ UTR regions of exons, the AD samples show a significant decrease compared to normal samples (*p*-value = 0.01), whereas the MCI samples exhibit no significant change (*p*-value = 0.55) (Figure S1).

**Splicing events and isoforms**: We identified a large number of alternative splicing events, with mutually exclusive exons, skipped exons, and retained introns being predominant in both the MCI and AD samples compared to the normal controls (Figure 2C). Among these splicing events, 3946 were observed in the MCI samples, and 6929 in the AD samples. And 2171 genes harboring alternative splicing events were identified in the MCI samples, and 2815 such genes were detected in AD samples (Table S1).

To explore the potential biological relevance of these splicing events, we conducted a pathway-enrichment analysis, which indicated that significantly different splicing events were involved in classical AD-related phenotypes, such as amyloid production. Furthermore, genes with the highest number of alternative splicing events were primarily associated with processes like cell polarity (e.g., microtubule transport) and oxidative stress response (e.g., lipid oxidation and proteasomal degradation of misfolded proteins) (Table S1).

**Differential expression analyses**: We performed differential expression analyses using the STAR pipeline, not distinguishing among different splicing isoforms for each gene. In the ROSMAP dataset, we identified 371 differentially expressed genes (DEGs) in the MCI samples and 1195 DEGs in the AD group (Figure 2D). We note that 35.04% of the DEGs are protein-coding genes in the MCI group and 45.27% in the AD group, while 64.96% and 54.73% of the DEGs are lncRNAs in the MCI and AD groups, respectively, and the rest are pseudogenes (Table 2).

To ensure the generalizability of these findings, we have conducted similar analyses on other AD datasets. The MSBB dataset exhibited a similar pattern to the one above, with 38.89% and 37.84% of DEGs being protein-encoding genes in early-stage and late-stage AD samples (Figure 2E). Similar results were observed in other brain regions within the MSBB dataset and in the GEO dataset GSE95587, as summarized in Figure S2 and detailed in Table 2.

**Figure 3:**
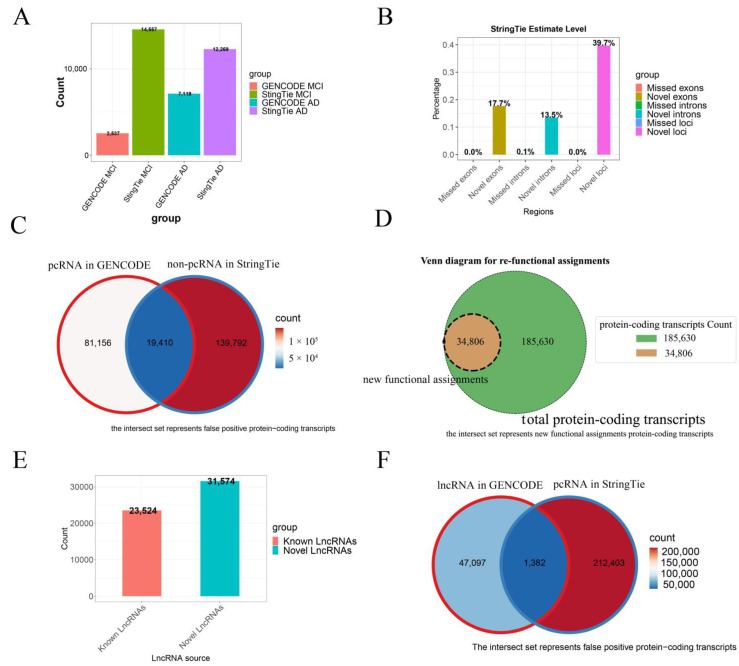
False positive and false negative rates of isoforms in the current transcriptomic datasets. (**A**) Proportions of differentially expressed protein-coding transcripts (DETs) in GENCODE and StringTie. The x-axis represents the source of the protein-coding transcripts, and the y-axis shows the proportion of each transcript type. (**B**) Improvement in identifying novel exonic, intronic, and intergenic regions using the optimized genome annotation file compared to the GENCODE genome. (**C**) False positives in protein-coding transcripts, where the overlap refers to transcript names found in both GENCODE and StringTie annotations. (**D**) False negatives in protein-coding transcripts, with the overlap indicating transcript names shared between the GENCODE database and StringTie annotation, but linked to different protein-coding transcripts. (**E**) Statistics on the number of known lncRNAs and newly assembled lncRNAs. (**F**) False positives in lncRNA transcripts, where the overlap refers to transcript names present in both GENCODE and StringTie annotations.

Overall, compared to existing transcriptomic datasets for AD tissues, our assembly significantly expands the coverage: we identified a 4.74-fold more (12,020 vs. 2537) differentially expressed protein-coding transcripts in MCI tissues, while in the AD samples, the coverage showed a slight decrease (5150 vs. 7119), representing a 0.72-fold decrease (Figure 3A).

**Pathway-enrichment analyses**: We then conducted pathway-enrichment analyses over the DEGs and found that the following were enriched: (1) amyloid production; (2) microtubule transport; (3) lipid oxidation; (4) proteasomal degradation of misfolded proteins (Table S2); (5) immune cell infiltration, including B cells and T cells, in the early-stage AD patients, which are typically absent in the brain (Table S2); and (6) surprisingly, no amyloid formation, a hallmark of early AD pathology, being detected in the enrichment results. This absence was consistently observed across multiple datasets (Table S2), suggesting that amyloid formation may not be as prominent at the transcriptomic gene level as previously assumed or that the changes are very subtle and dispersed across different isoform groups.

Our analyses have revealed that our assembled transcripts cover 17.7% more exonic regions, 13.5% more intronic regions, and 39.7% more intergenic regions when mapping our assembled transcripts to the reference genome in GENCODE (Figure 3B), hence all these regions being considered *novel*. The following provides a detailed analysis of our assembled transcripts and functional annotations:

**Statistics on the protein-coding transcripts:** Our analyses revealed that 19.3% (19,410 out of 100,566) of the existing transcripts annotated to be protein-coding were actually non-coding (Figure 3C), and 16.28% (34,806 out of 213,785) of the protein-coding transcripts are incorrectly assigned functionally (Figure 3D), where 100,566 is the number of protein-coding transcripts in the GENCODE dataset, and 213,785 represents the number of protein-coding transcripts identified by StringTie.

Furthermore, we discovered 39.7% more transcripts from the previously unannotated protein-coding genes, consisting of 17.7% novel exons and 13.5% novel introns (Figure 3B). Overall, we assembled 431,781 complete transcripts, corresponding to 213,785 protein-coding genes, out of which 178,979 transcripts have been functionally annotated.

**Statistics on LncRNAs:** We identified 55,098 lncRNAs, including 31,574 novel and 23,524 known ones (Figure 3E). Notably, 1382 originally annotated as lncRNAs have protein-coding potential (Figure 3F), hence actually being protein-coding transcripts, while none of the annotated protein-coding transcripts are annotated as lncRNAs by our study (Figure S3).

### 3.3. Applications of Our Assembled Transcripts

To verify that our assembled transcripts provide considerably more information about the development of AD than the existing transcripts, we focused on several key hallmarks of the disease: amyloid plaque formation [50], Tau fibril aggregation [51], extracellular acidosis [52], intracellular pH increase [5], and iron accumulation [50]. Additionally, we have also examined the functional roles played by lncRNAs in mediating metabolic reprogramming to alleviate extracellular acidosis and intracellular alkalosis. For the simplicity of discussion, we refer to the transcripts expressed in AD and currently available in the public domain as *the existing transcripts*, and our assembled transcripts using expressed RNA-seq reads in AD tissues as *our transcripts*.

**Figure 4:**
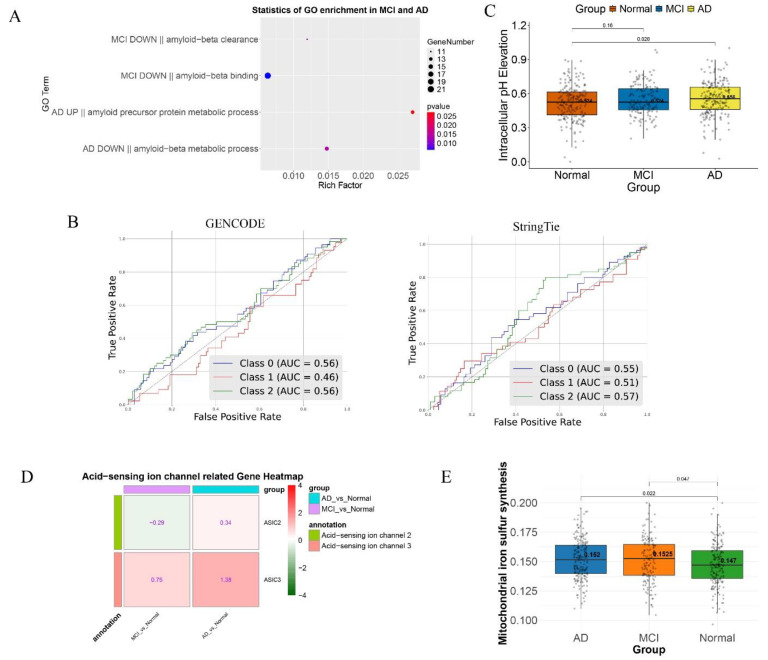
Functional validation of the new framework. (**A**) Enrichment results of amyloid formation-associated transcripts under the new framework. (**B**) AUC evaluation of amyloid formation-related transcripts using GENCODE and StringTie. (**C**) Evaluation of intracellular alkalosis under the new framework. (**D**) Assessment of extracellular acidosis under the new framework. (**E**) Evaluation of mitochondrial iron accumulation under the new framework.

**Amyloid plaque formation:** The existing transcripts consist of 774 amyloid-related transcripts vs. 854 such transcripts in our transcripts (Table 3). The existing transcripts expressed in the MCI group did not enrich the pathway of amyloid formation, while our transcripts enrich multiple related pathways such as decline in amyloid clearance capability and significantly elevated expression of APP precursor protein (Figure 4A). These differences enable us to accurately classify normal, MCI, and AD samples using amyloid-related transcripts (Figure 4B). Overall, our improved transcript dataset provides more information leading to deeper insights about the molecular mechanisms driving amyloid pathology, particularly in early stages of AD.

One example is that extracellular acidosis is a hallmark of AD and contributes to neuronal death. The accumulation of extracellular copper ions triggers Fenton reactions that generate OH^−^, which helps mitigate extracellular acidosis. Additionally, the hydroxyl radicals (˙OH) produced during these reactions promote the aggregation of Aβ into plaques. These amyloid plaques, in turn, form alkaline structures that further contribute to the progression of AD by stabilizing Aβ monomers and altering the extracellular environment [53].

**Tau fibril aggregation:** Our transcripts cover 347 Tau fibril-related transcripts, compared to 154 in the existing transcripts (Table 3), which gives more accurate classification among normal, MCI, and AD samples using such transcripts as shown in Figure S4. In addition, the significantly higher number of Tau fibril-related differentially expressed transcripts (DETs) enables more accurate understanding about the roles played by Tau hyperphosphorylation and Tau fibril aggregation in AD progression as shown in Figure S5.

A related example suggests that Tau hyperphosphorylation releases H^+^, helping mitigate intracellular pH elevation, while enriched pathways show that Tau fibril formation significantly suppresses synapse formation, leading to impaired neurotransmitter release and contributing to extracellular acidosis [53].

**Intracellular alkalosis and extracellular acidosis:** Intracellular alkalosis and extracellular acidosis are established features of AD [5,52]. Our transcripts consist of 10 more intracellular alkalosis-related transcripts than the existing transcripts (Table 3). These transcripts also exhibit significantly higher expressions in AD samples than the normal and MCI groups (Figure 4C), suggesting that intracellular alkalosis is more pronounced in AD than previously thought [40].

The expression levels of ASIC2 and ASIC3, widely used biomarkers for extracellular acidosis in our transcripts, are significantly higher in AD and MCI samples than in the normal samples, which are bot-detectable in the existing data (Figure 4D). This suggests that our transcripts provide more information enabling for detailed studies of pH dysregulation in AD.

**Iron accumulation and Fenton reactions:** Previous studies have reported persistent Fenton reactions in AD tissue cells [50] as follows:

$$O_{2}^{\cdot }+H_{2}O_{2}\;\overset{{\mathrm{Fe}}^{2+}}{\to }\;\cdot \mathrm{OH}\;+{\;\mathrm{OH}}^{-}+{\;O}_{2}$$

where ${\mathrm{Fe}}^{2+}$ serves as the catalyst, where iron accumulation is observed [54]. Our new transcripts reveal a significant increase in iron accumulation in AD (Figure 4E), covering 371 more iron-related transcripts, specifically 1265 vs. 904 in matching normal controls. Furthermore, we confirmed the availability of the other reactants of the Fenton reaction, namely the superoxide anion and hydrogen peroxide, based on our previous work [40] (Figure S6).

**Roles played by lncRNAs in pH regulation:** Our analysis of lncRNAs revealed their critical roles in keeping the intracellular pH stable, namely in response to intracellular alkalosis and extracellular acidosis. Compared to previous datasets, our assembled transcripts consist of 496 unique lncRNAs that are directly involved in pH-regulation pathways, a significant increase from the 119 lncRNAs documented in traditional datasets (Table 3). These transcripts were found to mediate key cellular processes related to pH balance, as outlined below:

1. Intracellular alkalosis: LncRNAs in our dataset promote mitochondrial iron accumulation, which in turn produces hydroxide (${\mathrm{OH}}^{-}$) and hydroxyl radical (OH) through Fenton reactions (Figure S7), causing intracellular alkalization. Specifically, 2471 lncRNAs are involved in regulating Fenton reactions. On the other side, lncRNAs are also involved in actions counterbalancing the alkalization via activating the acidification process. For example, lncRNAs MSTRG.44295.1 and MSTRG.14265.27 contribute to the activation of acid-loading transporters that lower the intracellular pH. Overall, our transcripts cover 646 lncRNAs vs. 309 lncRNAs in the existing transcripts relevant to intracellular pH (Table 3);
2. Extracellular acidosis: Our transcripts cover 812 vs. 229 lncRNAs by the existing transcripts related to regulating bicarbonate (HCO_3_^−^) transporters, which help to slow down the process of extracellular acidosis (Table 3).

We also note that in the early stage of AD, lncRNAs are involved in promoting the formation of amyloid plaques and Tau protein hyperphosphorylation. Our previous work has discovered that the formation of amyloid plaques slows down the process of extracellular acidosis since the folded structure of amyloid plaques tend to keep their alkaline sidechains pointing outwards while having their acidic sidechains folded inside the structure [40,53]. Similarly, we have also discovered that hyperphosphorylation on Tau proteins reduces intracellular alkalinity since each phosphorylation act releases one net proton [40,53]. Overall, our transcripts show over 990 lncRNAs involved in these two processes compared to 286 such lncRNAs in the existing transcripts.

Overall, our transcripts not only increase the number of relevant transcripts and enrich more related pathways but also improve the level of detail in AD-related mechanisms that can be studied. All phenotypes and related transcripts involved in this framework are listed in Table S3, and a comparison between the numbers of transcripts related to each of the key mechanisms in our transcripts and the existing ones are listed in Table 3. Additionally, Supplementary Data S3 presents the PCA results for all evaluated gene sets in both datasets, achieving a 60% variance explained.

## 4. Discussion

In this study, we constructed a transcriptomic dataset for AD and MCI tissues by assembling raw RNA reads in the public domain, which considerably expands the existing transcript dataset [16,55], enabling more in-depth and comprehensive studies of AD biology. Specifically, our data covers 178,979 protein-coding transcripts and 55,098 lncRNAs. In addition to this new and the state-of-the-art transcriptomic dataset, we have developed a computational pipeline for assembling full-length transcripts from raw RNA-seq reads and for functional annotation for each transcript through mapping it uniquely to a known protein sequence in humans or other organisms with known functions. Furthermore, a few analysis tools are also included in the pipeline, including pathway-enrichment analysis and prediction of the regulatory target genes or pathways of lncRNAs. By applying our transcript data and the computational pipeline, we have discovered:

1. **Correction of false positives and negatives in protein-coding transcripts**: A major contribution of our approach is the correction of numerous false positives and false negatives in the protein-coding transcript data for AD, which are prevalent in existing datasets. Using our pipeline, we identified and rectified these errors, resulting in a more accurate and reliable transcript catalog for AD research. For example, we detected 39.7% more protein-coding transcripts from previously unannotated genes. This correction is critical for ensuring the accuracy of downstream analyses and functional studies of AD-associated genes [16];
2. **LncRNA and pH imbalance**: The significantly increased number and improved quality of lncRNAs in our dataset have allowed us to further investigate the role of these molecules in AD pathogenesis, particularly in relation to pH regulation. Previous studies, including our own [40,53], have shown that pH imbalance plays a critical role in AD, with disruptions in pH homeostasis contributing to neuronal dysfunction. Our findings suggest that lncRNAs are involved in maintaining pH stability by regulating the expression of acidifying transporters. Specifically, we found that certain lncRNAs may help counteract intracellular alkalosis by activating H^+^-producing enzymes, which are central to maintaining pH balance in cells [56,57];
3. **Metabolic reprogramming in response to pH imbalance:** Our study also provides new insights into the metabolic reprogramming that occurs in AD as a result of pH imbalance. We observed that pH changes trigger a cascade of metabolic events, including the activation of cytosolic acidifying transporters, to counteract the effects of intracellular alkalosis and extracellular acidosis [40,53]. This metabolic reprogramming is essential for maintaining cellular homeostasis in AD, and our data highlight key pathways involved in pH regulation that could potentially be targeted for therapeutic intervention. Specifically, H^+^-producing enzymes play a critical role in this process, supporting the idea that targeting these enzymes may help correct pH imbalances in AD neurons and alleviate disease progression [58].

Despite these advancements, several limitations must be considered. First, our reliance on postmortem brain tissue may not fully capture the dynamic changes occurring in the earlier stages of AD [59], and rare or low-abundance transcripts may remain undetected despite improved transcript coverage. Additionally, while our findings highlight pathways involved in pH regulation, key signaling pathways such as mTOR and NOTCH, which are central to AD pathogenesis, warrant further investigation [60,61]. mTOR plays a pivotal role in cellular metabolism and protein homeostasis, with dysregulation linked to impaired autophagy, amyloid-β accumulation, and tau hyperphosphorylation [62,63]. Similarly, NOTCH is involved in neurodegeneration through its regulation of amyloid precursor protein processing and its contribution to tau pathology via phosphorylation and aggregation [64]. Both pathways may also interact with pH regulatory mechanisms, such as the modulation of lysosomal function and proton transporters, highlighting their potential as therapeutic targets in AD [65,66]. Further exploration of these mechanisms could provide valuable insights into AD progression and opportunities for intervention.

In summary, our assembled transcriptomic dataset represents a significant advance in AD research, enabling new tools and insights that could enhance the understanding of disease mechanisms and improve the study of MCI and AD biology.

## 5. Conclusions

Transcriptomic data have proven to be the most powerful type of omic data for disease studies in terms of derivation of their drivers and key molecular mechanisms. Our study has demonstrated that the currently available transcriptomic data for AD and pre-AD tissues in the public domain are far from being adequate for detailed studies of the disease. Our new data source has proven to be significantly more informative than the existing one for AD studies.

## Figures and Tables

**Table 1:** Summary of assembled transcript.

Cohort	Region	Stage	#Samples	#Protein-Encoding Transcripts	#LncRNAs
ROSMAP	Dorsolateral prefrontal cortex	Normal	189	206,573	49,965
ROSMAP	Dorsolateral prefrontal cortex	MCI	146	204,800	49,259
ROSMAP	Dorsolateral prefrontal cortex	AD	193	206,175	49,988

**Table 2:** Composition statistics of differential gene types across various AD datasets.

Cohort	Region	Group	Gene Types	Number	Percentage
ROSMAP	Dorsolateral prefrontal cortex	MCI	lncRNA	154	0.415
ROSMAP	Dorsolateral prefrontal cortex	MCI	protein-coding	130	0.350
ROSMAP	Dorsolateral prefrontal cortex	MCI	other non-coding RNA	87	0.235
ROSMAP	Dorsolateral prefrontal cortex	AD	lncRNA	516	0.432
ROSMAP	Dorsolateral prefrontal cortex	AD	protein-coding	541	0.453
ROSMAP	Dorsolateral prefrontal cortex	AD	other non-coding RNA	138	0.115
MSBB	Superior temporal gyrus	CDR I-II	lncRNA	261	0.110
MSBB	Superior temporal gyrus	CDR I-II	protein-coding	1067	0.448
MSBB	Superior temporal gyrus	CDR I-II	other non-coding RNA	1055	0.443
MSBB	Parahippocampal gyrus	CDR I-II	lncRNA	491	0.123
MSBB	Parahippocampal gyrus	CDR I-II	protein-coding	1671	0.417
MSBB	Parahippocampal gyrus	CDR I-II	other non-coding RNA	1844	0.460
MSBB	Inferior frontal gyrus	CDR I-II	lncRNA	506	0.116
MSBB	Inferior frontal gyrus	CDR I-II	protein-coding	1741	0.399
MSBB	Inferior frontal gyrus	CDR I-II	other non-coding RNA	2119	0.485
MSBB	Superior temporal gyrus	CDR III-IV	lncRNA	1206	0.146
MSBB	Superior temporal gyrus	CDR III-IV	protein-coding	3395	0.411
MSBB	Superior temporal gyrus	CDR III-IV	other non-coding RNA	3666	0.443
MSBB	Parahippocampal gyrus	CDR III-IV	lncRNA	1356	0.117
MSBB	Parahippocampal gyrus	CDR III-IV	protein-coding	5927	0.513
MSBB	Parahippocampal gyrus	CDR III-IV	other non-coding RNA	4273	0.370
MSBB	Inferior frontal gyrus	CDR III-IV	lncRNA	976	0.121
MSBB	Inferior frontal gyrus	CDR III-IV	protein-coding	2649	0.329
MSBB	Inferior frontal gyrus	CDR III-IV	other non-coding RNA	4436	0.550
GSE95587	Fusiform gyrus	Braak III-IV	lncRNA	316	0.257
GSE95587	Fusiform gyrus	Braak III-IV	protein-coding	722	0.587
GSE95587	Fusiform gyrus	Braak III-IV	other non-coding RNA	191	0.155
GSE95587	Fusiform gyrus	Braak V-VI	lncRNA	323	0.211
GSE95587	Fusiform gyrus	Braak V-VI	protein-coding	754	0.492
GSE95587	Fusiform gyrus	Braak V-VI	other non-coding RNA	456	0.297

**Note:** CDR (Clinical Dementia Rating): a standard used to assess the severity of dementia of patients. Braak staging is a system for measuring the progression level of AD based on neuropathological changes in the brain.

**Table 3:** The number of transcripts associated with AD hallmarks in our vs. the existing transcripts.

Phenotype	Our Protein-Coding Transcripts	The Existing Protein-Coding Transcripts	Our LncRNA Transcripts	The Existing Lncrna Transcripts
Intracellular alkalosis	125	115	496	119
Extracellular acidosis	39	28	605	310
Mitochondrial iron accumulation	1265	904	819	340
Amyloid formation	854	774	833	286
Tau fiber formation	347	154	1077	306
Superoxide generation	314	376	825	333
Hydrogen peroxide generation	213	186	827	324
Acid-loading transporter	182	117	646	309
Bicarbonate transporter	146	122	812	229

## Data Availability

The data supporting the reported results can be found at https://drive.google.com/drive/folders/17CWxRNx1Cdm17IIb_d8_lyg12VrRohTx (accessed on 21 November 2024).

## Supplementary Materials

The following supporting information can be downloaded at: https://www.mdpi.com/article/10.3390/brainsci14121180/s1 or https://drive.google.com/drive/folders/17CWxRNx1Cdm17IIb_d8_lyg12VrRohTx (accessed on 21 November 2024). Figure S1: 3‘UTR alignment rates across normal, MCI, and AD groups; Figure S2: Statistics of the number of protein-coding differential expression transcripts in GSE95587 and the other brain regions in MSBB cohort; Figure S3: No predicted protein-coding RNAs display LncRNA features; Figure S4: AUC evaluation of Tau fiber formation-related transcripts using GENCODE and StringTie; Figure S5: Increased number of Tau fibril-related DETs in AD progression; Figure S6: Comparison of the levels of hydrogen peroxide generation across three groups. The x-axis is for groups, and the y-axis for the level of hydroxyl radicals. The data are presented as median ± standard deviation (SD) for each group. B. Comparison of superoxide anion generation levels across three groups; Figure S7: A. The numbers of lncRNAs involved in mitochondrial iron sulfur clustering synthesis across the four bins. B. The numbers of lncRNAs involved in superoxide anion generation across the four bins. C. The numbers of lncRNAs involved in superoxide anion generation across the four bins; Table S1: Genes harboring alternative splicing events in MCI and AD samples; Table S2: Pathway-enrichment analyses of differentially expressed genes (DEGs) in AD progression; Table S3: All phenotypes and related transcripts involved in this framework; Supplementary Data S1: New lncRNA sequences and genome annotations, including the genomic locations of all transcripts, and protein-coding transcripts with their corresponding gene names; Supplementary Data S2: Differential transcripts and enriched pathways; Supplementary Data S3: PCA-explained variance statistics for the gene set regulated by lncRNAs.
