# Supplementary material for: Construction of a Dataset for All Expressed Transcripts for Alzheimer’s Disease Research
Source: Brain Sci. 2024 Nov 25;14(12):1180. doi: 10.3390/brainsci14121180 (PMC11674848; doi:10.3390/brainsci14121180)
Supplement: Supplementary file 1 [file brainsci-14-01180-s001.zip › Supplementary Information.pdf]

SUPPLEMENTARY MATERIAL FOR

Construction of A Dataset for All Expressed Transcripts for Alzheimer's Disease Research

Zhenyu Huang[1,2], Bocheng Shi[2,3], Xuechen Mu[2,3], Siyu Qiao[2], Gangyi Xiao[1], Yan Wang[1*], Ying Xu[2*]
[1]College of Computer Science and Technology, Jilin University, Changchun, China, [2]Systems Biology Lab for Metabolic Reprogramming, Department of Human Genetics and Cell Biology, School of Medicine, Southern University of Science and Technology, Shenzhen, China, [3]School of Mathematics, Jilin University, Changchun, China.
*Correspondence: wy6868@jlu.edu.cn, xuy9@sustech.edu.cn

The PDF file includes:

Supplementary Tables: Table S1 to S3
Supplementary Data: Data S1 to S3
Supplementary Figures: Figure S1 to S7

A. **Supplementary Tables**

Table S1: Genes harboring alternative splicing events in MCI and AD samples.

Table S2: Pathway enrichment analyses of differentially expressed genes (DEGs) in AD progression.

Table S3: All phenotypes and related transcripts involved in this framework.

B. **Supplementary Data**

Supplementary Data S1: New lncRNA sequences and genome annotations, including the genomic locations of all transcripts, and protein-coding transcripts with their corresponding gene names.

Supplementary Data S2: Differential Transcripts and Enriched Pathways;

Supplementary Data S3: PCA explained variance statistics for the gene set regulated by lncRNAs.

C. **Supplementary Figures**

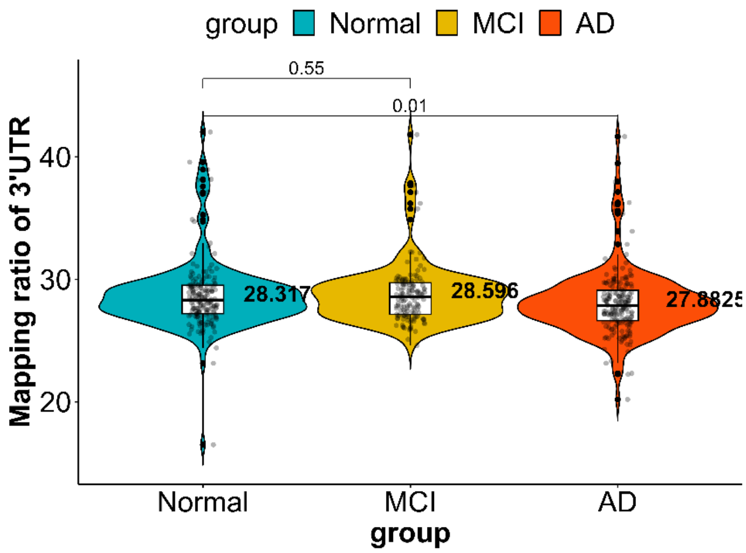

Figure S1. 3'UTR alignment rates across Normal, MCI, and AD groups.

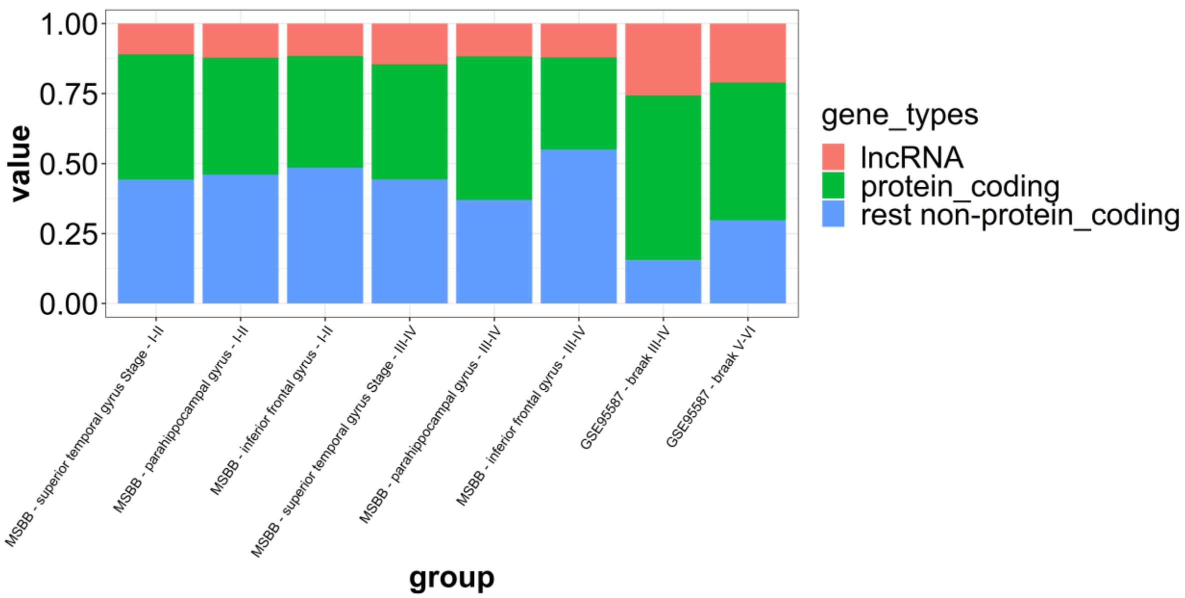

Figure S2. Statistics of the number of protein-coding differential expression transcripts in GSE95587 and the other brain regions in MSBB cohort.

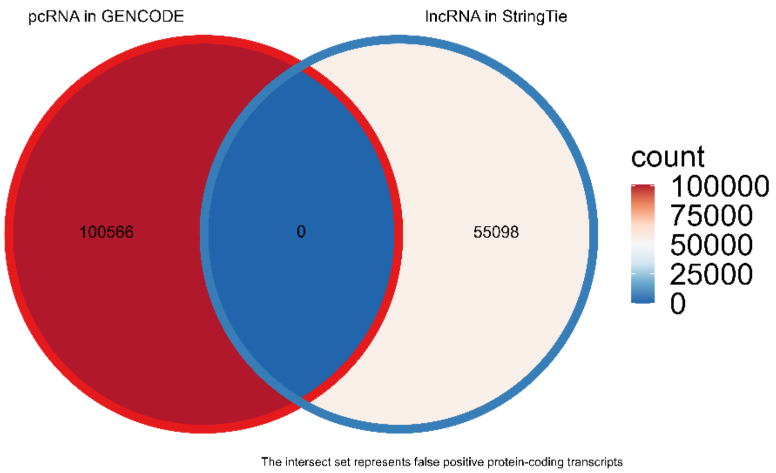

Figure S3. No predicted protein-coding RNAs display LncRNA features

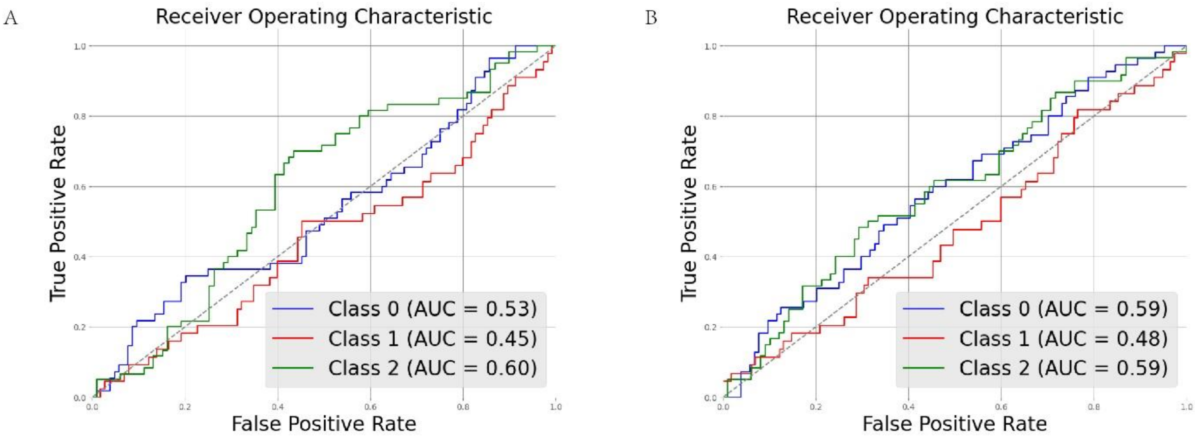

Figure S4. AUC evaluation of Tau fiber formation-related transcripts using GENCODE and StringTie.

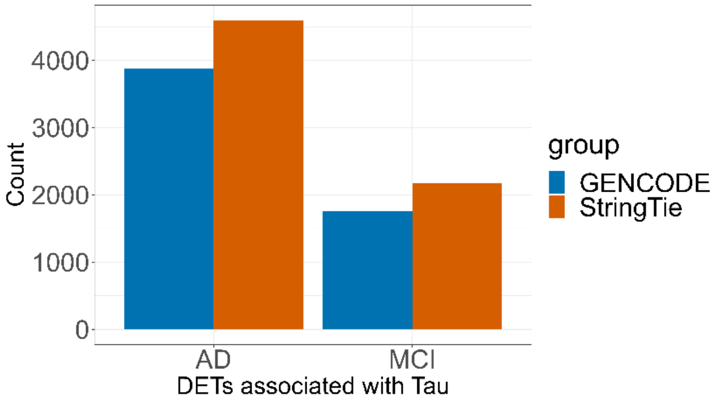

Figure S5. Increased number of Tau-fibril related DETs in AD progression.

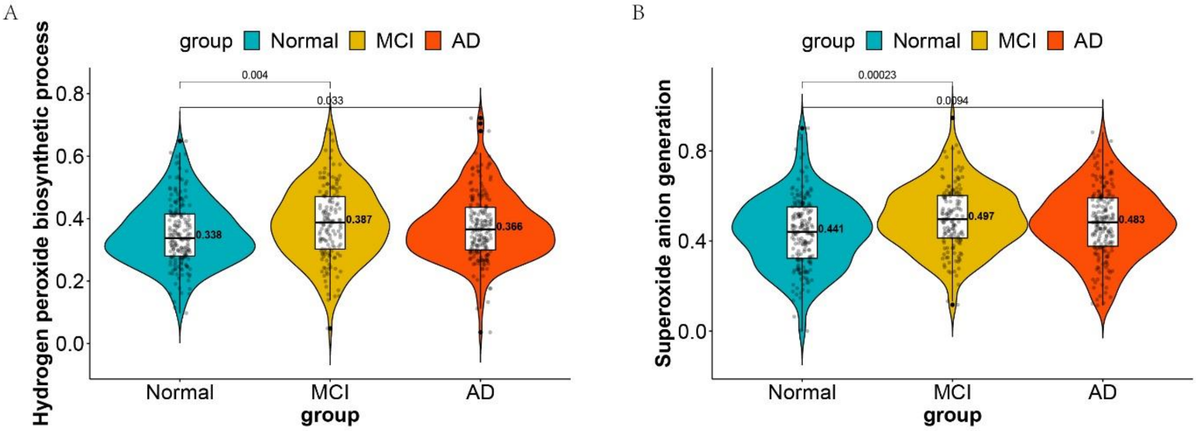

Figure S6. A. Comparison of the levels of hydrogen peroxide generation across three groups. The x-axis is for groups, and the y-axis for the level of hydroxyl radicals. The data are presented as median ± standard deviation (SD) for each group. **B.** Comparison of superoxide anion generation levels across three groups.

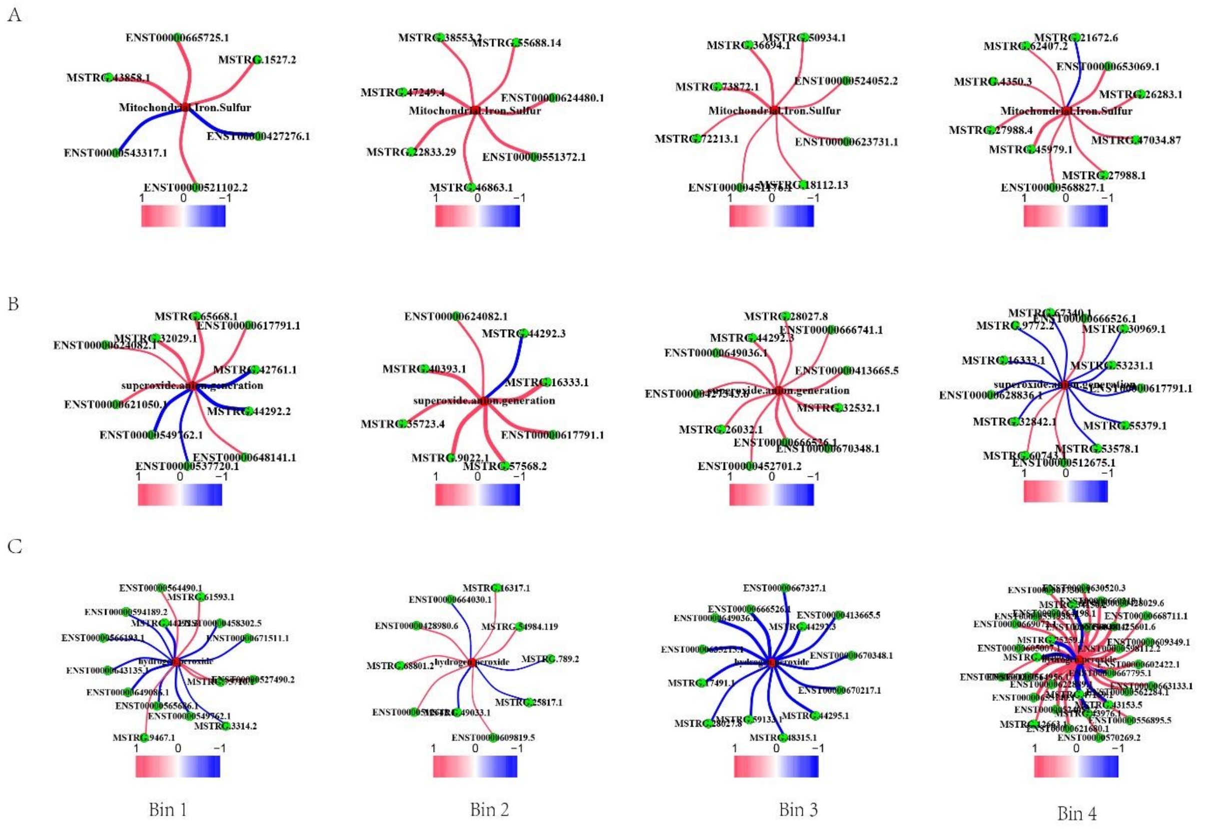

Figure S7. A. The numbers of lncRNAs involved in mitochondrial iron sulfur clustering synthesis across the four bins. **B.** The numbers of lncRNAs involved in superoxide anion generation across the four bins. **C.** The numbers of lncRNAs involved in superoxide anion generation across the four bins.
